# Is the Dog a Possible Reservoir for Cutaneous Leishmaniasis in Suriname?

**DOI:** 10.1155/2013/324140

**Published:** 2013-10-01

**Authors:** Alida Kent, Prakash Ramkalup, Dennis Mans, Henk Schallig

**Affiliations:** ^1^Anton de Kom University of Suriname, Paramaribo, Suriname; ^2^Department of Parasitology, Faculty of Medical Sciences, Kernkampweg 5, Paramaribo, Suriname; ^3^Veterinary Clinic Eerste Rijweg, Paramaribo, Suriname; ^4^Royal Tropical Institute, Amsterdam 1105 AZ, The Netherlands

## Abstract

Cutaneous leishmaniasis (CL) is an emerging disease in Suriname, with at least 200 cases per year. Little is known about the biology of CL in the country. The most important parasite species is *Leishmania Viannia guyanensis*, but possible vectors and reservoirs are hardly incriminated. In the present study, it was investigated whether the dog could possibly be a zoonotic reservoir for the disease in Suriname. Forty-seven dogs were examined for overt clinical signs of leishmaniasis, and blood samples were collected on filter paper for serology (direct agglutination test) and molecular biology (by polymerase chain reaction). Three dogs had clinical signs that could be compatible with canine cutaneous leishmaniosis: dermatitis (two) or nasal lesion (one). Two dogs were seropositive with DAT (titre > 1 : 1600), and three animals had a borderline titre (1 : 800). All other animals (*n* = 42) were DAT negative. PCR analysis found *Leishmania* DNA equivalent to 1 parasite per mL in only one dog at a first round of analysis, but this animal was negative after retesting. The clinical, serological, and molecular data show some preliminary lines of evidence that canine leishmaniosis is present in Suriname, but further studies are needed to incriminate the reservoir, including a possible sylvatic cycle.

## 1. Introduction

Cutaneous leishmaniasis (CL) is a skin disease ranging from self-healing lesions to single or large skin ulcers and is caused by protozoan parasites of the genus *Leishmania. *CL is a zoonotic disease transmitted via the bite of female sand flies belonging to the genera *Phlebotomus* in the Old World and *Lutzomyia* in the New World [1]. Humans are most likely only incidental hosts. In countries where zoonotic leishmaniasis occurs—such as those in Latin America and those around the Mediterranean basin—dogs are considered the main domestic reservoirs for human infection [2]. Dogs can also be severely affected by the infection, and canine leishmaniosis (CanL) can present as a systemic disease with as main clinical manifestations lymphadenopathy, dermatitis, alopecia, cutaneous ulcerations, onychogryphosis, lameness, anorexia, weight loss, cachexia, ocular lesions, epistaxis, anaemia, diarrhoea, and renal failure [3]. Furthermore, CanL can also present with symptoms and signs similar to CL in humans. The signs and symptoms of canine CL include skin lesions that begin with the formation of a nodule at the place where the bite occurred. This nodule increases in size and then may ulcerate. Lesions may also look as plaques. The lesions usually occur on the nose, ears, and perineal region that are the body regions where dogs have less hair [4]. Importantly, a significant proportion of infected animals remain asymptomatic [5], but these asymptomatic infected canines may act as carriers of *Leishmania* and are capable of transmitting the parasite to sand flies [6].

Early diagnosis and treatment of dogs has proven effective to stop transmission to other dogs and humans in several endemic countries and could be part of disease control programs [3]. 

In Suriname, CL represents an increasingly important public health problem, with a mean annual detection rate of 5.32 to 6.13 CL patients per 1,000 inhabitants for the forested interior and 0.64 to 0.74 patients per 1,000 inhabitants for the whole country [7]. The disease is mainly contracted in the forested interior of the country and is locally known as bosyaws or busi yasi. *Leishmania (V.) guyanensis *is the principle species causing CL in Suriname, but recently other species have also been identified, including *L (V.) braziliensis* [8], *L (V.) naiffi* [9], and *L (L.) amazonensis* [7]. Treatment is with pentamidine isethionate, which is the first-line medication for infections with *L. (V.) guyanensis *and at present the only available antileishmanial drug in the country. 

So far, a possible animal reservoir for CL in Suriname has not unambiguously been identified [10]. However, there is an increasing trend to regard dogs also as important domestic reservoirs for CL in this country and other countries of the New World [11]. Notably, in the interior of Suriname, dogs are commonly kept by local communities and gold miners for hunting and to guard residences. Furthermore, individuals from Suriname's capital city Paramaribo go regularly on hunting trips to the interior of the country with their dogs. Consequently, both urban and rural canines may experience a significant risk of contracting this disease.

Based on these considerations, the present observational study was conducted to investigate whether the dog could represent a zoonotic reservoir for CL in Suriname. To this end, a population of dogs from various parts of the country was evaluated by clinical observation and standard serological and molecular methods for signs and symptoms of CanL [3, 12–16]. The results obtained may provide indications as to whether the dog represents a possible reservoir for CL in Suriname. 

## 2. Materials and Methods

### 2.1. Dogs and Collection of Blood Samples

The dogs were examined and sampled with routine procedures. The clinical examinations did not affect animal welfare. Sacrifice of animals did not occur. Blood sampling is a routine procedure and does not cause excessive harm or pain to the animals. The animals were examined by qualified veterinarians. Dog owners were asked to participate in the study when they had to have their animals examined.

The dogs that were evaluated in this study could be assigned to four groups. Group 1 (*n* = 10) comprised dogs from the animal shelter in Paramaribo (Stichting Dierenbescherming Suriname). It was not known whether or not these dogs had been taken to the interior of the country where CL is prevalent. Group 2 (*n* = 11) included animals from Paramaribo which had never been brought outside the urbanized regions of Suriname. Group 3 (*n* = 8) were hunting dogs from Paramaribo that were regularly taken on trips to the interior of the country, thus having an increased likelihood of being exposed to sand flies. Group 4 (*n* = 18) comprised dogs from emerging CL foci in Suriname, namely, Afobaka and Witagron.

The animals were clinically examined by veterinary practitioners and considered either apparently healthy or clinically suspect when either none, or at least one clinical sign or lesion compatible with CanL, respectively, was noted. Suspicious signs or lesions included local or general lymphadenopathy, dermatitis, alopecia, cutaneous ulcerations, onychogryphosis, lameness, weight loss, ocular or nasal lesions, epistaxis, and anemia. Blood samples were collected from a vein in the leg and placed in sterile, EDTA-coated tubes subsequent to spotting in the middle of a filter paper. They were allowed to air-dry and stored at –20°C until analysis [17]. 

### 2.2. Serology

*Leishmania*-specific antibodies in the blood samples were determined using the direct agglutination test (DAT) [12, 14]. The samples were analyzed with DAT based on freeze-dried antigen (batch: 1212), produced by The Royal Tropical Institute, Amsterdam, The Netherlands, with a cut-off titre > 1 : 400 to maximize sensitivity and specificity of the test [12, 14]. A DAT titre of 1 : 800 is considered borderline, and a serum with a DAT titre of ≥ 1 : 1.600 is considered positive [17].

For testing, one filter paper disk of 5.5 mm in diameter was punched out from the middle of the spot and eluted overnight at 4°C in 0.9% saline to produce the equivalent of a 1 : 100 serum dilution. Next, the serum samples were diluted in physiological saline (0.9% NaCl) containing 1.56% *β*-mercaptoethanol and supplemented with fetal calf serum (1%). Twofold dilution series were made from 1 : 100 to 1 : 102,400 in V-shaped microtitre plates (Greiner, Germany) and incubated 1 h at 37°C. Fifty microliters of reconstituted DAT antigen were subsequently added to each well containing 50 *μ*L diluted serum. Quantitative results obtained with DAT are expressed as an antibody titre, that is, the reciprocal of the highest dilution at which agglutination (large diffuse blue mats) is still visible after 18 h incubation at room temperature. 

Serum samples of dogs from Portugal with a by culture, DNA detection, and serology-confirmed leishmaniosis infection (DAT titre > 1 : 6.400) were used as positive controls for serology. As negative controls, sera with a DAT titre < 1 : 100 from healthy dogs from a nonendemic country, The Netherlands, were used.

### 2.3. Molecular Biology

#### 2.3.1. DNA Extraction

DNA was isolated from bloodspots on filter paper according to the protocol described by Boom et al. [18]. In brief, a filter paper disk of 5.5 mm diameter was punched out from the middle of a blood spot and incubated with 1 mL L6 lysis buffer (50 mM Tris HCl, 5 M GuSCN, 20 mM EDTA, 0,1% Triton-X-100) overnight at room temperature. Next, the supernatant was collected and mixed for 5 min with 30 *μ*L silica gel (SiO_2_, Sigma S5631) to trap the DNA and centrifuged for 15 sec at 12,000 g. The silica pellet was collected and washed repeatedly with L2 wash buffer (50 mM Tris HCl (Boehringer), 5 M guSCN (Fluka), 70% ethanol, and acetone). DNA was eluted in 50 *μ*L TE buffer (Tris EDTA buffer, 100x concentrated Sigma) and stored at −20°C until analysis.

#### 2.3.2. Polymerase Chain Reaction (PCR)

The DNA samples were analyzed by real-time PCR specific for all *Leishmania *species [19], with some slight modifications. Briefly, each amplification reaction contained 2.5 *μ*L of isolated DNA sample and was added to 22.5 *μ*L amplification mix containing 1x master mix (BioRad), 0.8 *μ*M of each primer (18S Forward primer and 18S Reverse primer), and 0.2 *μ*M 6-carboxyfluorescein (FAM) taqman probe. Amplification and real-time measurements were performed in the BioRad opticon minicycler, with the following conditions: 10 min at 50°C, 5 min at 95°C followed by 45 cycles of 30 sec at 95°C, and 45 sec at 60°C. The samples were compared to a 10-fold *L. donovani* DNA dilution series ranging from 10 to 10^7^ parasites per reaction for possible quantification and served as a positive amplification control. Water controls were included to exclude possible cross-contaminations.

## 3. Results

### 3.1. General Characteristics of the Dogs Assessed in This Study

A total of 47 dogs was examined during the different sampling rounds. Forty-five animals were of mixed breed and two were Rottweilers. Twenty-three dogs were female (54.8%) and 19 were male (45.2%), while the gender of five dogs was not determined. The mean age of the animals was 29 months (range 6 months–14 years). Three dogs showed some clinical features that could be compatible with CanL. Two of them had dermatitis, but this was most likely caused by a myiasis (“maskita woron”). The remaining animal (a male bastard of 18 months from Afobaka) had a nasal lesion which could be considered as a typical clinical manifestation of cutaneous CanL. All other examined dogs showed no overt clinical signs of CanL.

### 3.2. DAT Titres of *Leishmania*-Specific Antibodies

The DAT analyses were all valid, as positive and negative controls reacted according to their predetermined status, and the titers of *Leishmania*-specific antibodies of appropriate control samples determined with DAT corresponded with the pre-determined titer of the control sera.

In total, 35 dogs had a DAT titre ≤ 1 : 100, two had a titre of 1 : 200, and five had a titre of 1 : 400. All of them (*n* = 42; 89.4%) could be considered seronegative for CanL. Three animals had a borderline titre of 1 : 800. Two of them originated from Paramaribo and one from Witagron. 

Two dogs had a DAT titre of 1 : 1.600 and could be considered seropositive for canine leishmaniosis. The first dog was a 2-year-old female from Witagron with no apparent clinical signs of CanL. The second animal was sampled in Afobaka and was a male of 1-2 years of age, also without clinical signs.

### 3.3. Detection of *Leishmania *DNA

**Figure 1:**
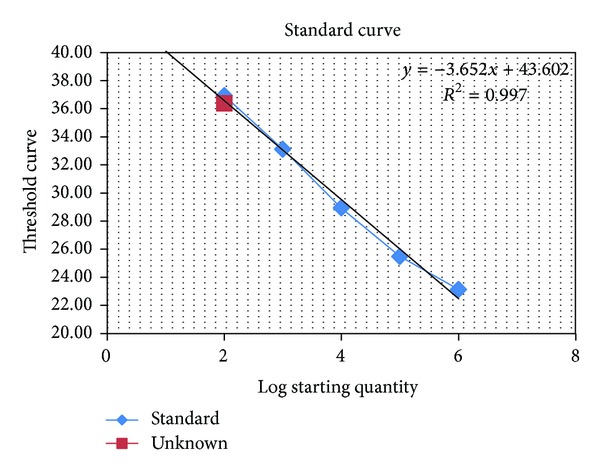
Standard curve obtained with qPCR on serial dilutions of *Leishmania* DNA (Log Starting Quantity) ranging from 10 to 10^7^ parasites per reaction, with signal of the positive sample (unknown: red square) indicated.

All dog samples were analyzed twice by real-time PCR, and all but one were negative in the two rounds of testing. One sample tested positive the first time with a parasite count of 1 parasite/mL (Figure 1), but it was negative when repeated. This sample was from a 3-year-old female bastard from the animal shelter in Paramaribo. Interestingly, this animal had a DAT titre of 1 : 800, but no clinical signs of canine leishmaniosis.

## 4. Discussion

The zoonotic reservoir for CL in Suriname is still unknown. However, in other countries in the Americas, evidence is accumulating that dogs and other domestic animals may serve as reservoirs in foci of CL [4]. Furthermore, such animals may attract vectors to domestic sites, increasing the risk of infection of humans [11]. The species known to cause CL in dogs are *L (V.) braziliensis, L (V.) panamensis,* *L (V.) guyanensis*, and *L (V.) peruviana* [4, 20]. *L (V.) braziliensis* and *L (V.) guyanensis* are known to cause CL in Suriname and are present in high-transmission areas in the country [7, 8].

The current study provides some lines of evidence for the presence of canine cutaneous leishmaniosis in Suriname. *Leishmania* DNA was detected in one dog, albeit only once and around the threshold of the molecular test employed [19]. However, this is not surprising as the parasite load of *Leishmania Viannia* species in blood is low [21, 22]. Furthermore, this dog had a borderline DAT titre of 1 : 800. This animal was sampled in the kennel in Paramaribo, and its whereabouts are unknown. Dogs are brought to the shelter from all over the country, and no information is available about past residences.

Furthermore, two dogs were found seropositive for canine leishmaniosis. The first dog was sampled in Witagron and the second in Afobaka, and both locations are well-known endemic foci for CL in Suriname. *Leishmania *DNA could not be detected in these animals. However, this might be attributable to past infections that had been controlled by the dogs' immune system [22].

All the three dogs lacked the clinical signs associated with canine leishmaniosis [3]. However, as canine cutaneous leishmaniasis is often asymptomatic, the absence of lesions does not necessarily signify the absence of the disease [22]. 

Taken together, the clinical observations (three dogs with some signs), serological findings (two seropositive animals), and molecular data (DNA detected in one dog) may suggest that canine leishmaniosis is present in Suriname, but further studies are needed to substantiate this assumption.

During the different sampling rounds, dog owners appeared very reluctant to have their animals examined and sampled for CanL. As a result, the sample size of the current study turned out rather small. The reluctance to participate in such studies is probably attributable to a lack of awareness of the risks of CanL to human health. Such an attitude has also been observed in other countries such as Portugal, where dogs must be treated if found positive for the disease [17]. However, since the costs for the treatment are substantial, many owners hide their animals when a survey is being performed [17]. An effective option to circumvent this problem is to screen dogs during mandatory anti-rabies vaccination campaigns [17]. 

In addition to dogs, many other possible reservoir animals for leishmaniasis have been identified. These could either be domestic, such as cats [23] and horses [24], or part of the wild life in a so-called sylvatic cycle. In particular in South and Central America, not only *Leishmania* species are known to affect humans, but sylvatic species such as *L. forattinii*, *L. enrietti*, and *L. deanei* have been encountered in wild animals such as tree-porcupines [25], foxes (*Dusicyon vetulus* and *Cerdocyon thous*) [26, 27], rodents [28, 29], and opossums (*Didelphis marsupialis*) [30–32]. Future studies on incriminating possible reservoirs for leishmaniasis in Suriname should therefore also consider the fauna of the interior.

## 5. Conclusion

The present study provides the first indication for the possible occurrence of canine leishmaniosis and a possible role of canines in the transmission of cutaneous leishmaniasis in Suriname. This observation emphasizes the need for more targeted studies in the CL hotspots of the country to monitor dogs and local fauna for possible infections with *Leishmania* sp. Such surveys would generate important knowledge to further develop a control program for leishmaniasis in Suriname as it must include measurements to interrupt the sylvatic cycle and require the identification of potential reservoir animals.
